# Novel Honokiol-eluting PLGA-based scaffold effectively restricts the growth of renal cancer cells

**DOI:** 10.1371/journal.pone.0243837

**Published:** 2020-12-17

**Authors:** Yasaman Hamedani, Samik Chakraborty, Akash Sabarwal, Soumitro Pal, Sankha Bhowmick, Murugabaskar Balan

**Affiliations:** 1 Department of Mechanical Engineering, Biomedical Engineering and Biotechnology Program, University of Massachusetts Dartmouth, Dartmouth, MA, United States of America; 2 Division of Nephrology, Boston Children’s Hospital, Boston, MA, United States of America; 3 Harvard Medical School, Boston, MA, United States of America; Ohio State University, UNITED STATES

## Abstract

Renal Cell Carcinoma (RCC) often becomes resistant to targeted therapies, and in addition, dose-dependent toxicities limit the effectiveness of therapeutic agents. Therefore, identifying novel drug delivery approaches to achieve optimal dosing of therapeutic agents can be beneficial in managing toxicities and to attain optimal therapeutic effects. Previously, we have demonstrated that Honokiol, a natural compound with potent anti-tumorigenic and anti-inflammatory effects, can induce cancer cell apoptosis and inhibit the growth of renal tumors *in vivo*. In cancer treatment, implant-based drug delivery systems can be used for gradual and sustained delivery of therapeutic agents like Honokiol to minimize systemic toxicity. Electrospun polymeric fibrous scaffolds are ideal candidates to be used as drug implants due to their favorable morphological properties such as high surface to volume ratio, flexibility and ease of fabrication. In this study, we fabricated Honokiol-loaded Poly(lactide-co-glycolide) (PLGA) electrospun scaffolds; and evaluated their structural characterization and biological activity. Proton nuclear magnetic resonance data proved the existence of Honokiol in the drug loaded polymeric scaffolds. The release kinetics showed that only 24% of the loaded Honokiol were released in 24hr, suggesting that sustained delivery of Honokiol is feasible. We calculated the cumulative concentration of the Honokiol released from the scaffold in 24hr; and the extent of renal cancer cell apoptosis induced with the released Honokiol is similar to an equivalent concentration of direct application of Honokiol. Also, Honokiol-loaded scaffolds placed directly in renal cell culture inhibited renal cancer cell proliferation and migration. Together, we demonstrate that Honokiol delivered through electrospun PLGA-based scaffolds is effective in inhibiting the growth of renal cancer cells; and our data necessitates further *in vivo* studies to explore the potential of sustained release of therapeutic agents-loaded electrospun scaffolds in the treatment of RCC and other cancer types.

## Introduction

Solid tumors of the kidney are collectively known as Kidney Cancer or Renal Cell Carcinoma (RCC); and it is among the 10 most frequently diagnosed cancers in the United States [1]. Based on histological and genomic profiles, clear cell RCC (ccRCC) and papillary RCC (pRCC) are the two major subtypes of RCC, and chromophobe RCC is a rare one [1]. Although the overall survival rate has increased due to advancement in tumor detection and treatments, the five-year survival rate is only 8% among patients with metastatic RCC [1]. Renal cancer cells are dependent on Receptor Tyrosine Kinases (RTK) like c-MET for their survival and RTK targeted therapeutics (like, sorafenib, sunitinib, cabozantinib and others) are being used in the first-line of treatment of RCC [2–4]. However, treatment options for advanced RCC are limited and, certainly, metastatic RCC is a major clinical problem and is aggressive [4]. Thus, novel therapeutic agents are needed. In addition, dose-dependent toxicity is a major hurdle in the clinical use of RTK targeted therapeutics (like c-MET inhibitor cabozantinib), subduing the anti-tumor effects of RTK inhibitor therapy [3]. Hence, equally important is to explore new methods to deliver anti-tumor agents to mitigate dose-dependent toxicity. Our recent reports suggest that Honokiol exhibits anti-tumor properties against renal cancer cells and, therefore, studying novel delivery methods of Honokiol can be significant in exploring Honokiol as novel treatment of RCC [5, 6].

Honokiol (C_18_H_18_O_2_) is a bi-phenolic lignan, naturally present in different parts of Magnolia plant, with potent anti-inflammatory and pro-apoptotic properties [7]. Several reports, including ours, demonstrate the anti-tumor properties of Honokiol in different cancer types [5, 6, 8–10]. Honokiol treatment induces cancer cell apoptosis through different mechanisms including activation of caspases, suppression of anti-apoptotic proteins Bcl-2 and Bcl-XL and inhibition of cyto-protective HO-1 [5]. With its anti-inflammatory properties, Honokiol can also be used as an adjunct immunosuppressive agent for transplant patients to achieve immune suppression in preventing transplant organ rejection and, in addition, it can potentially prevent the development of post-transplantation cancer in immunosuppressed patients [5]. However, the multi-therapeutic influence of Honokiol would be optimally achieved only if its suitable concentration is supplied either systemically or to the diseased area with the ability of sustained release. Thus, exploring novel methods for proper delivery of Honokiol is crucial.

A variety of drug delivery systems have been studied so far; namely: polymeric micelles, liposomes, nanoparticles, hydrogels, bioactive membranes, and electrospun nanofibers [11–17]. Strategies for drug encapsulation and release can range from simple loading in a polymeric carrier and providing release through a combination of diffusion, surface hydrolysis and degradation of the matrix or a more controlled approach using stimuli responsive (pH, redox, oxidation and enzyme concentration) or remote controlled (magnetic field, temperature, light intensity and ultrasound frequency) systems [18, 19].

Electrospinning is a micro/nanofabrication technique in which application of electrostatic force results in obtaining continuous fine fibers from polymeric solutions. Electrospun nanofibrous scaffolds have been widely used as controlled drug delivery systems due to their tunable submicron architecture, which can encapsulate drugs and release them by various mechanisms [20–22]. Moreover, versatility in choice of polymeric composition and design of the whole scaffold architecture, such as single layer vs sandwich-structure multi layers, or microscopic architecture at individual fiber scale, such as solid fibers or core-shell ones, bring about the potential of manipulating the delivery in a desired trend. The release trends can be classified as immediate, sustained, controlled or pulsatile [23–28]. Drug loaded nanofibrous patches can be implanted into the desired area in the body to provide a source of the drug and local delivery of the therapeutic agent. Proper design of the micro-architecture would engineer the delivery to the diseased organ in a controlled fashion. This will eliminate the need to inject the drug in multiple times to keep the therapeutic concentration, which provides patient comfort and reduces toxicity potential. Specifically, in case of cancer treatment, drug-loaded implants not only provide a local and efficient delivery but also minimize the systemic toxicity [29]. Likewise, local delivery of Honokiol using electrospun scaffolds for RCC treatment, would eliminate the potential toxicity problems associated with direct injection.

In this study, we successfully demonstrated the enclosure of Honokiol into electrospun Poly (lactide–co–glycolide)(PLGA) nanofibers, an FDA approved synthetic polymer frequently used as a biocompatible drug carrier. We monitored the morphology of the produced fibers and chemically characterized them to ensure the preservation of the drug’s chemical structure. We investigated the release behavior of the loaded Honokiol, as well as it’s biological activity *in vitro*, in inducing apoptosis and inhibiting the proliferation and migration of renal cancer cells. The results were compared to the direct application of Honokiol in the same therapeutic concentration, on the same cell types.

## Materials and methods

### Polymers and reagents

Poly Lactide -co-glycolide acid (PLGA) Resomer LG 857 was purchased from Boehringer Ingelheim company, 2,2,2-Trifluoroethanol (TFE) >99%, was purchased from Sigma Aldrich company. Synthetized pure Honokiol (Molecular Weight 266.334) was purchased from Sellekchem (Houston Texas). Primary antibodies against Bcl-2, Bcl-xL, HO-1 and species-specific secondary antibodies were obtained from Cell Signaling. Antibody against β-actin was purchased from Sigma Aldrich.

### Cell lines

Human renal cancer cell lines (786-O and ACHN) were obtained from ATCC. 786-O and ACHN cell lines were maintained in RPMI 1640 medium. All medium were supplemented with 10% fetal bovine serum (FBS) and 1% penicillin-streptomycin antibiotics (Invitrogen).

### Electrospinning

A home-built electrospinning device was used consisting of a Glassman high voltage power supply (EH-Series), Braintree scientific syringe pump (BS-8000) and a flat aluminum plate covered with a thin aluminum foil. All the different parts were enclosed in an environmental controlled chamber capable of providing various percentage of relative humidity (16% up to 90%). PLGA with Lactic acid: Glycolic acid ratio of 85–15 was dissolved in 2,2,2-Trifluoroethanol (TFE) solvent to achieve proper spinnable concentration. The polymeric solutions with or without Honokiol with drug to polymer weight ratio of 0.2, were placed inside of a 1 mL Hamilton glass syringe connected to a flat tip 22-gauge metal needle. The syringe was placed inside of the flow pump, 17 cm away from the stationary aluminum deposition collector, which was covered by a thin Aluminum foil. The flow rate of the solution was set at 0.2 mL/hr. The applied voltage to the tip of the needle was 15 kV. The collector was completely grounded. The deposition time was monitored accurately to have samples with the exact thicknesses. Various electrospinning parameters were optimized to obtain continuous bead free fibers.

### Scanning Electron Microscopy (SEM)

The morphology of the Electrospun samples was monitored by Hitachi Scanning Electron Microscopy SU5000. Briefly the samples were placed on the double-sided carbon tape, mounted on the Hitachi Scanning Electron Microscopy stubs and monitored at different accelerating voltage of 5–20 kV. The average fiber diameters were measured using NIH ImageJ software.

### Proton nuclear magnetic resonance spectroscopy (HNMR)

The fabricated scaffolds, with and without Honokiol, as well as the Honokiol powder were dissolved in deuterated chloroform at a concentration of 10 mg/mL and loaded in Bruker ADVANCE III HD 400 MHz High-Performance Digital NMR for their 1H NMR spectra. The obtained spectra were analyzed using MestreNova software.

### Drug release assay

The drug-loaded fibrous electrospun mats were removed from the foil and used for drug release assay. For this purpose, scaffolds with the same dimensions (0.5x0.5 cm^2^) and thicknesses were placed in 200 μL release medium (Phenol red free RMPI media with pH 7.2) in 96 well plate and kept at 37 C and 6% CO2. In different time intervals; 5 minutes until 48 hours, the release medium was collected. The collected media was analyzed using a UV-Vis equipped plate reader at 254 nm.

### Apoptosis assay

Cellular apoptosis was measured using an allophycocyanin (APC)-conjugated Annexin-V and propidium iodide (PI) apoptosis detection kit (Thermo Fisher Scientific, Waltham, MA). Following staining, the cells were analyzed by flow cytometry on a FACS Calibur.

### Cell proliferation assay

MTT cell proliferation assay (Thermo Fisher Scientific, Waltham, MA) kit was used to quantify cell proliferation, following manufacturer’s protocol. Briefly, 10,000 cells/well were seeded in 96 well plate. Following treatments, media was removed, and 10μl of MTT dye was added to each well. Cell proliferation assay was performed using the MTT kit according to manufacturer’s protocol.

### Western blot

Cells were lysed and protein samples were resolved on SDS-polyacrylamide gels and transferred to a polyvinylidene difluoride membrane (Milipore corporation). The membranes were incubated with primary antibodies and subsequently incubated with peroxidase-linked secondary antibodies. Reactive bands were detected using chemiluminescent substrate (Pierce) on X-ray films.

### *In vitro* wound healing assay

Cells were seeded on 6-well culture plates. When cells were grown to confluence, medium was aspirated and, using a 200 μL pipette tip, cells were scratched along the diameter of the well, to simulate the wound. Cells were washed twice with PBS and either 0.5x0.5 cm^2^ Honokiol-loaded scaffold or control was added in serum-free medium as indicated. At 0 hr and 12 hr after incubation, wound diameter in each well was photographed using phase contrast microscope to assess wound closure. Number of cells migrated to the wound area beyond the reference line were quantified using NIH ImageJ software.

### Statistical analysis

Statistical significance was determined by Student’s t test. Differences with p < 0.05 were considered statistically significant.

## Results

### Morphological characterization of PLGA and Honokiol loaded PLGA fibers

**Fig 1:**
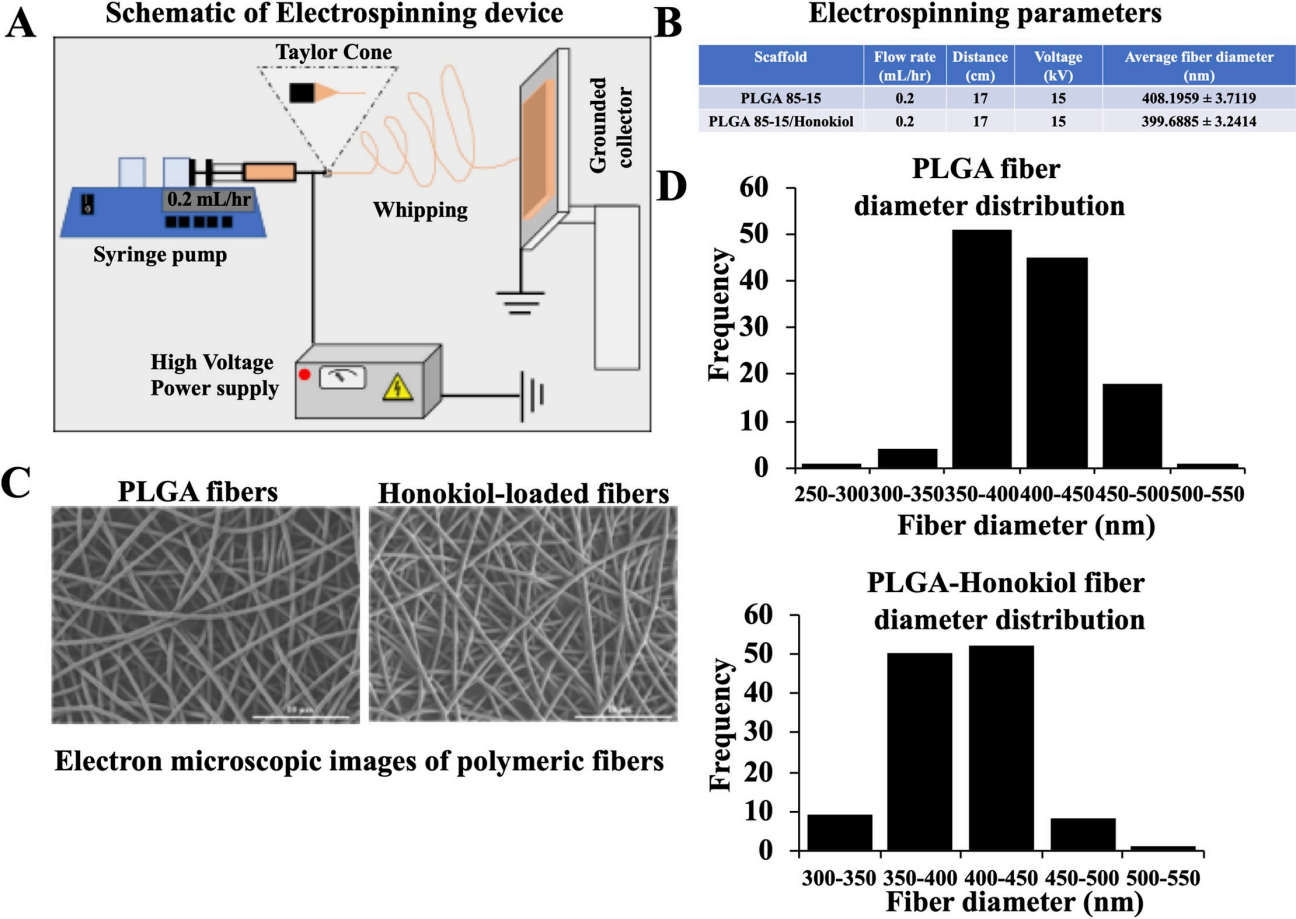
Morphological characterization of control and Honokiol loaded PLGA 85–15 fibers. (A) Schematic representation of the electrospinning process. (B) Summary of the optimized parameters used in the electrospinning process to fabricate continuous bead free fibers. (C) Scanning Electron Microscope pictures of control PLGA 85–15 (*left*), and Honokiol-loaded PLGA 85–15 electrospun scaffold (*right*). (D) Fiber diameter distribution of PLGA 85–15 (*top*) and Honokiol loaded PLGA 85–15 scaffolds (*down*).

Honokiol loaded PLGA 85–15 fibers can be used to achieve slow and sustained delivery of the drug. To obtain the Honokiol loaded PLGA 85–15 fibers, we utilized a custom-built electrospinning device, as schematically shown in Fig 1A. The electrospinning device consists of three different parts; flow pump, high voltage source and the grounded collector. In this method, the PLGA 85–15 polymeric solution is placed inside of a glass syringe in the syringe pump and the solution is pushed outside of the flat capillary with a specific flow rate. When high voltage is applied to the tip of the capillary, the drop is stretched until a point where the electrostatic forces balance out the surface tension of the drop, forming a cone at the tip of the needle, known as Taylor cone. When the electrostatic force overcomes the surface tension, a fiber jet accelerates towards the grounded collector, leaving the solvent evaporated as it passes through the distance between the needle and the collector. The fiber becomes thinner while undergoing whipping motions and eventually is solidified and deposited on the collector. We optimized the process and solution parameters to obtain continuous bead free fibers using 2 wt% PLGA 85–15 solution with and without Honokiol in a drug/polymer weight ratio of 0.2. Fig 1B summarizes the optimized electrospinning parameters used to fabricate continuous bead free fibers as well as the average fiber diameter of each scaffold. The continuous bead free fibers could be obtained when using 0.2 mL/hr flow rate, 17 cm distance and 15 kV voltage. Fig 1C shows the scanning electron microscopy (SEM) images of the electro-spun polymeric fibers with and without Honokiol, respectively. Fig 1D depicts the fiber diameter distribution of the scaffolds with and without the drug. The fiber diameter of PLGA 85–15 scaffold had almost stayed constant between 350 to 450 nm after enclosure of the drug and the average diameter was around 400 nm.

### Preservation of Honokiol chemical structure in electrospun scaffolds

HNMR analysis was performed to show the perseverance of Honokiol chemical structure after electrospinning process in the produced fibers. The chemical shifts of the Honokiol powder, PLGA 85–15 fibrous scaffolds without the drug and Honokiol-loaded PLGA 85–15 fibrous scaffolds are listed below:

❖ Honokiol:
^1^H NMR (400 MHz, Chloroform-*d*) δ 7.18–7.10 (m, 1H), 7.08 (s, 1H), 6.96 (d, *J* = 8.2 Hz, 1H), 5.97 (ddt, *J* = 16.9, 10.0, 6.7 Hz, 1H), 5.30 (s, 1H), 5.15–5.00 (m, 2H), 3.37 (d, *J* = 6.7 Hz, 2H).
❖ PLGA 85–15 fibers:
^1^H NMR (400 MHz, Chloroform-*d*) δ 5.16 (q, *J* = 7.1 Hz, 2H), 4.93–4.62 (m, 1H), 1.58 (dd, *J* = 7.2, 3.5 Hz, 6H).
❖ Honokiol loaded PLGA 85–15 fibers:
^1^H NMR (400 MHz, Chloroform-*d*) δ 7.14 (d, *J* = 7.7 Hz, 2H), 7.08 (s, 1H), 6.96 (d, *J* = 8.3 Hz, 1H), 5.97 (td, *J* = 16.7, 6.5 Hz, 1H), 5.40 (s, 0H), 5.16 (q, *J* = 7.2 Hz, 7H), 5.07 (d, *J* = 9.8 Hz, 1H), 4.92–4.63 (m, 2H), 4.06 (t, *J* = 6.6 Hz, 1H), 3.37 (d, *J* = 7.0 Hz, 2H), 1.58 (d, *J* = 7.2 Hz, 18H).

**Fig 2:**
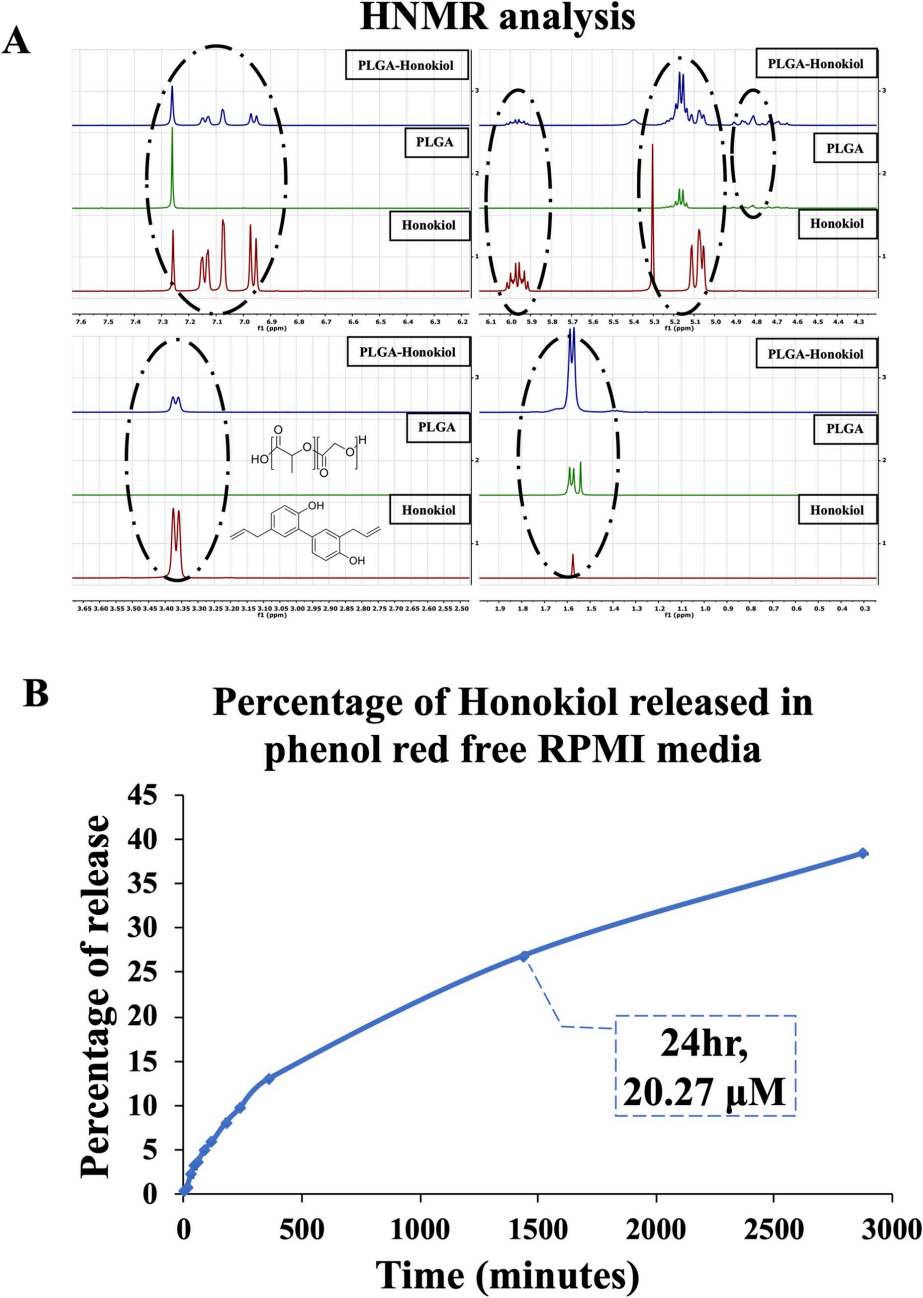
Preservation of the chemical structure of Honokiol after enclosure into PLGA 85–15 scaffolds. (A) HNMR spectra of Honokiol powder, PLGA 85–15, and Honokiol-loaded PLGA 85–15 scaffolds are presented. The peaks corresponding to Honokiol-specific proton resonance were circled to highlight the preservation of Honokiol chemical structure in the scaffold. (B) Using conditioned medium collected after incubation with 0.5x0.5 cm^2^ strips of either Honokiol-loaded scaffold or control at different time points, concentrations of Honokiol was measured by UV measurements, and plotted to obtain the pattern of the Honokiol released from the drug-loaded scaffolds. We found that about 20 μM of Honokiol was released from the scaffolds in 24 hr, corresponding to 24% of all the Honokiol loaded in the scaffold. Based on this release pattern, a 0.5x0.5 cm^2^ strip of Honokiol-loaded scaffold was used to achieve 20 μM of Honokiol in other *in vitro* experiments.

As shown in Fig 2A, the protons resonating at δ 7 ppm correspond to the aromatic ring of Honokiol can be seen both in Honokiol powder spectra and Honokiol-loaded PLGA 85–15 fibers. The protons resonating at 5.97 ppm and 5.30 ppm seen in both Honokiol powder and Honokiol-loaded PLGA 85–15 fibers correspond to carbon double bonds in Honokiol structure. The shift at 5.00 and 5.15 ppm are related to aliphatic structure, the protons resonating at 3.37 ppm are related to OH bonds in Honokiol and Honokiol loaded PLGA 85–15 scaffolds. Thus, we confirmed the perseveration of the chemical structure of Honokiol after enclosure into PLGA 85–15 scaffolds.

### *In vitro* release behavior of Honokiol released from Honokiol-loaded PLGA 85–15 scaffold and the biological activity of the released Honokiol

**Fig 3:**
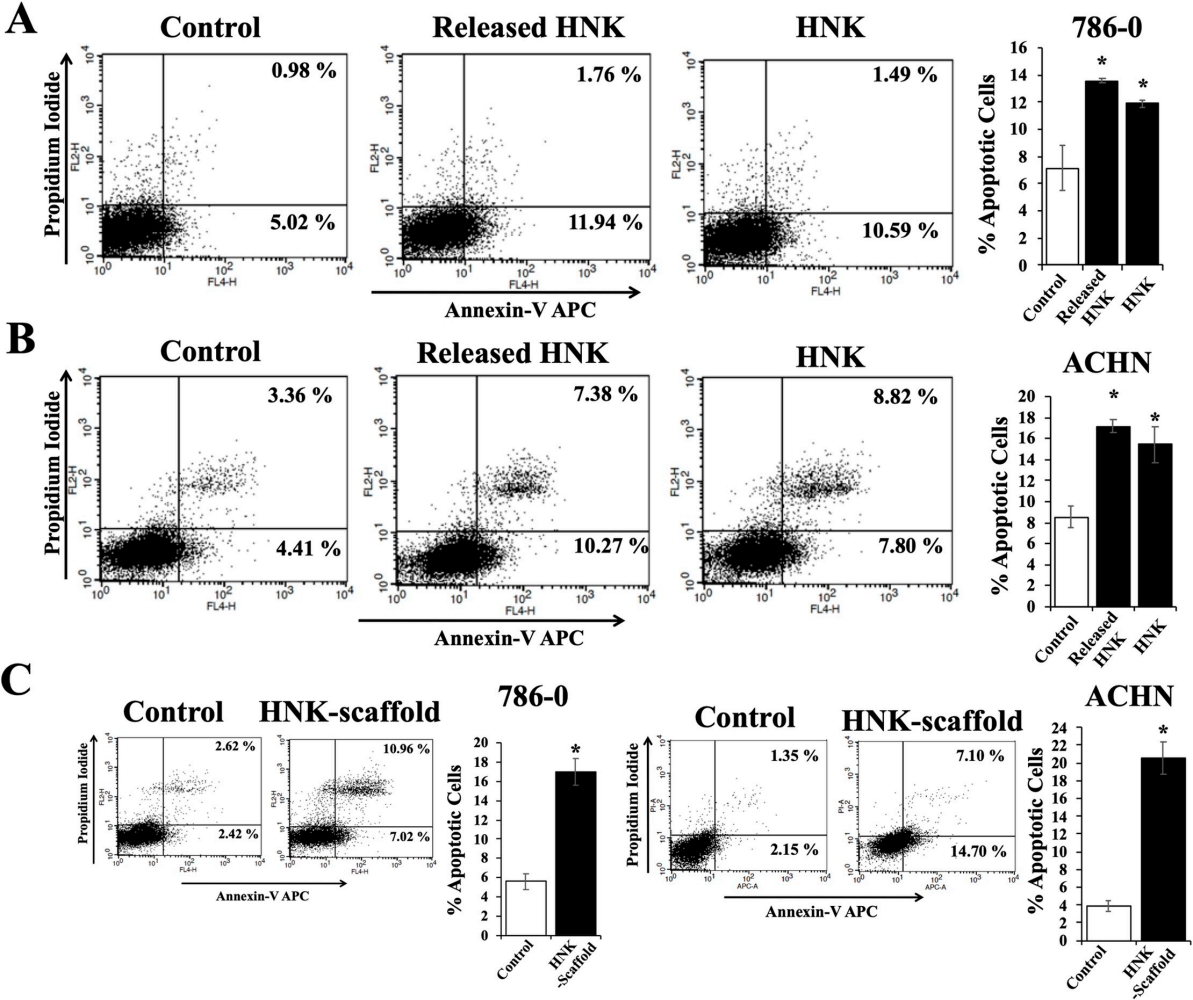
Apoptosis-inducing activity of Honokiol released from Honokiol-loaded PLGA 85–15 scaffold. (A) and (B) Renal cancer cells (786–0 and ACHN respectively) were treated with either the released Honokiol (20 μM) or Honokiol (20 μM) along with control for 24 hr. Following treatment, cells were stained with Annexin-V-APC and propidium iodide and the apoptotic indices were analyzed by flow cytometry. (C) Similarly, apoptotic indices of 786–0 and ACHN cells cultured with either 0.5x0.5 cm^2^ Honokiol-loaded PLGA 85–15 scaffold or control scaffold for 24 hr were analyzed. The bar graph presented next to the flow cytometry dot-plots represents the percentage of total apoptotic cells (early + late). A, B, and C results shown are representative of three independent experiments. The columns in the bar graphs represent the mean ± S.D. of experimental readings. *, represents p<0.05 compared with respective controls.

Here, we checked the release pattern of Honokiol from the drug loaded scaffolds. By performing UV measurements of the medium released after incubation with drug loaded scaffolds, we found that up to 24% of the Honokiol was released from a 0.5x0.5 cm^2^ scaffold after 24 hr, which corresponds to 20 μM cumulative Honokiol concentration (Fig 2B). To understand if the cell growth-induced pH changes of the cell culture medium affect the concentration of the Honokiol released from the scaffolds, we also incubated 0.5x0.5 cm^2^ of drug loaded scaffold directly into cell culture (cells were grown in phenol red free RPMI growth medium), and analyzed the cumulative Honokiol concentration in the culture medium; and it reached 20 μM after 24 hr (data not shown), comparable to the honokiol release pattern when scaffolds were incubated in culture medium without cells in the culture. Next, we evaluated whether the Honokiol released into the medium from the scaffolds is biologically active. Our recent reports establish that Honokiol induces apoptosis of renal cancer cells [5, 6] and therefore we tested the apoptotic effects of the Honokiol released from the scaffolds. Therefore, we cultured renal cancer cells (786–0 and ACHN) with the addition of calculated amount of conditioned medium collected after incubating 0.5x0.5 cm^2^ of Honokiol-loaded PLGA 85–15 scaffolds to achieve a final concentration of 20 μM or control medium, and assessed the apoptotic indices of cells after 24 hr. We also incubated the renal cancer cells with 20 μM of Honokiol for 24 hr, as a control. As shown in Fig 3A, we found that the incubation with Honokiol-loaded scaffolds markedly increased 786–0 renal cancer cell apoptosis; the total apoptotic cells (early + late) increased from (5.02 + 0.98) = 6.00% (control cells) to (11.94 + 1.76) = 13.7% (Honokiol loaded scaffold-treated cells). Importantly, the apoptotic indices of renal cancer cells treated with the high concentration of 20 μM for 24 hr (10.59 + 1.49) = 12.08% were comparable with that of the cells treated with the Honokiol released from the scaffolds (13.7%). We observed similar effects on ACHN cells (Fig 3B); and the total apoptotic cells increased from (4.41 + 3.36) = 7.46% (Control) to (4.00 + 5.00) = 17.65% (released Honokiol). We also checked the biological activity of the Honokiol-loaded scaffolds when directly placed in the cell culture medium. We cultured renal cancer cells (786–0) with the addition of 0.5x0.5 cm^2^ of either Honokiol-loaded PLGA 85–15 scaffolds or control scaffolds and assessed the apoptotic indices of cells after 24 hr. We found that the total apoptotic cells increased from (2.42 + 2.62) = 5.04% (control) to (7.02 + 10.96) = 17.98% (Honokiol-scaffolds) in 786–0 cells and it increased from (2.15 + 1.35) = 3.50% (control) to (14.70 + 7.10) = 21.80% (Honokiol-scaffolds) in ACHN cells, suggesting that the Honokiol released directly from the scaffold, effectively induced apoptosis of renal cancer cells (Fig 3C). Thus, Honokiol-loaded PLGA 85–15 scaffolds are suitable for gradual and sustained release of Honokiol, and at the same time the released Honokiol is biologically active.

### Released Honokiol inhibits the proliferation and migration of cancer cells and down-regulates the expression of intracellular molecules associated with the growth of renal cancer cells

**Fig 4:**
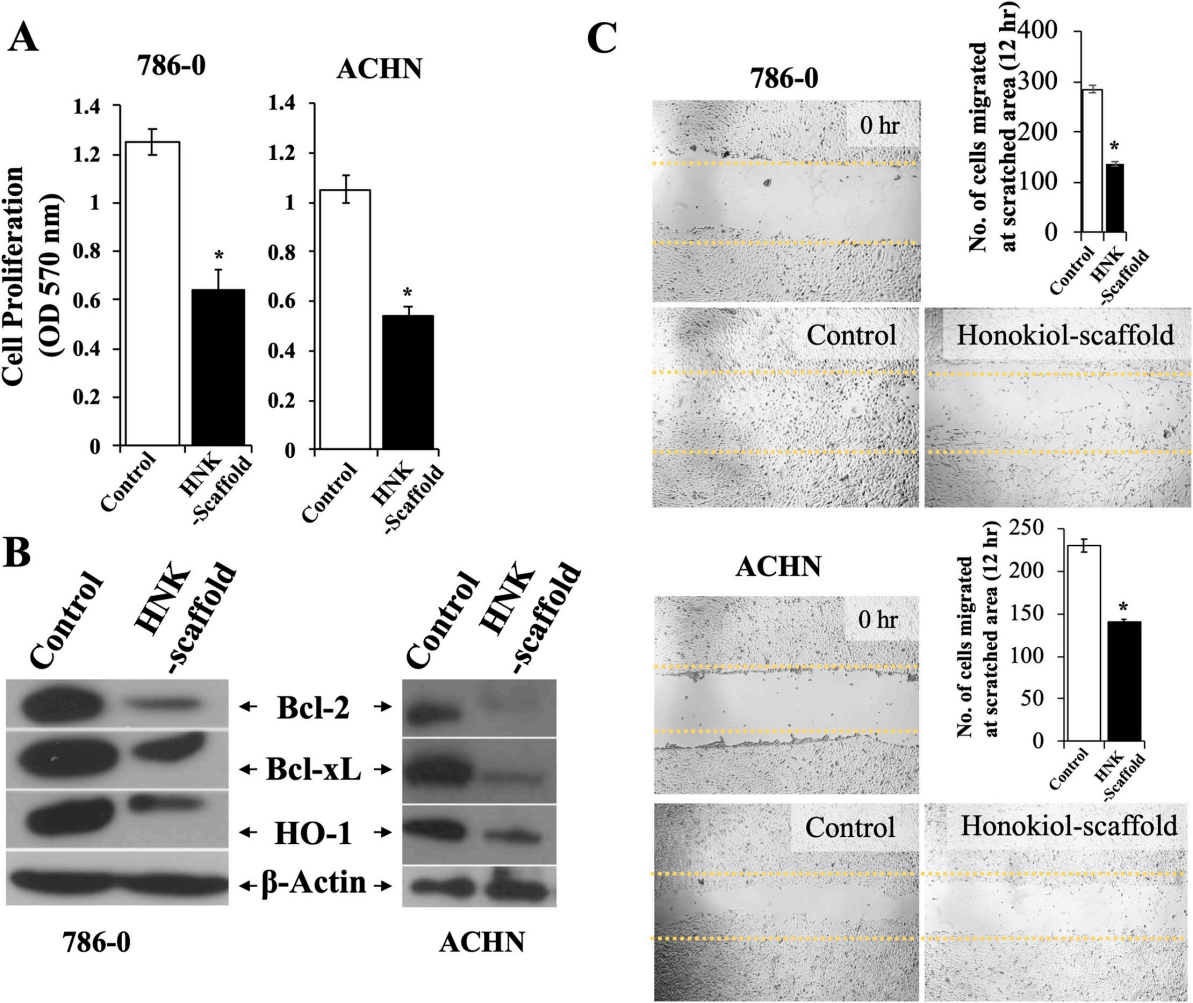
Biological activities of Honokiol released from Honokiol-loaded PLGA 85–15 scaffold. (A) Renal cancer cells (786–0 and ACHN) were treated with either 0.5x0.5 cm^2^ Honokiol-loaded PLGA 85–15 scaffold or control scaffold for 24 hr. Following treatment, cell proliferation was measured by MTT assay and (B) cell lysates were prepared after 24 hr treatment and the expression levels of Bcl-2, Bcl-xL, HO-1 and β-actin were analyzed by Western blotting. (C) “Wound healing assay”, as described in the “Methods” section was performed using 786–0 and ACHN cells to assess the migration of renal cancer cells. Briefly, cells were treated with either Honokiol-loaded PLGA 85–15 scaffold or control after scratching the diameter of the culture well with a 200 μl pipette tip to simulate a wound. To assess wound closure, the phase contrast microscope photographs of the wound area at 0 hr and after 12 hr treatment, were analyzed. The bar graph represents the quantification of number of cells migrated to the wound area. B, and C results shown are representative of three independent experiments. A-C, the columns in the bar graphs represent the mean ± S.D. of experimental readings. *, represents p<0.05 compared with respective controls.

As we observed that the gradual release of Honokiol from scaffold is active as equally as the direct addition of Honokiol, in inducing apoptosis of renal cancer cells, we further studied its biological effect in regulating the proliferation and migration of renal cancer cells. Correlating with our earlier reports [5, 6], we found that the released Honokiol significantly inhibited the proliferation of renal cancer cells (786-O and ACHN) when renal cancer cells were cultured with Honokiol-loaded scaffold compared with control (Fig 4A). We have reported that Honokiol-induced renal cancer cell apoptosis is associated with the decreased expression of anti-apoptotic proteins Bcl-2 and Bcl-xL and cytoprotective HO-1; and we checked whether the released Honokiol can modulate the expression of these intracellular molecules [5, 6]. As shown in Fig 4B, we observed that the released Honokiol significantly downregulated the expression of Bcl-2, Bcl-xL and HO-1 in renal cancer cells (786–0 and ACHN). In a similar experiment, the released Honokiol also inhibited the migration of renal cancer cells as measured by wound healing assay using 786–0 and ACHN cells (Fig 4C). Thus, the Honokiol released from the scaffolds can inhibit the proliferation and migration of renal cancer cells. Importantly, it can downregulate the expression of effector molecules associated with cancer cell proliferation and promote renal cancer cells apoptosis. Together, we demonstrate that Honokiol-loaded scaffolds can release biologically active Honokiol *in vitro*.

## Discussion

In this study, we prepared and characterized Honokiol-loaded PLGA 85–15 scaffolds. We achieved sustained release of Honokiol, which stays biologically active after being loaded into electrospun scaffolds. With the use of 786–0 and ACHN renal cancer cell lines, we have demonstrated that the released Honokiol can inhibit the proliferation and migration of renal cancer cells and promote cancer cell apoptosis *in vitro*.

In order for the drug to have proper therapeutic activity, it needs to reach the target in a suitable concentration and for an appropriate duration of time. Conventional dose-delivery of therapeutic agents require frequent administration to provide the proper therapeutic dosage, above the minimum therapeutic level and below the minimum toxic level [30]. However, bioavailability of the drugs and dose-dependent side effects are some of many limitations of the traditional drug delivery methods, like oral or intravenous injections [30–35]. To overcome these limitations, controlled drug delivery systems are being explored in different applications, specially cancer treatment. Controlled delivery of therapeutic agents such as Paclitaxel, Methotrexate, Stattic, Doxorubicin, and Cisplatin have been demonstrated with the use of PLGA-based nanoplatforms, silica nanoparticles, and pendant-dendron amphiphilic copolymer (P71D3)-based polymeric micelles and high tumor concentration of the therapeutic agent can complement controlled and targeted delivery through conjugating the therapeutic agents with tumor targeting antibodies or peptides [14–17, 36].

Aside from controlled drug delivery systems which require injection to the tumor area, implant-based controlled drug delivery systems, have the advantage of providing a controlled but also localized delivery of the drug and can minimize systemic toxicity. Electrospun PLGA scaffolds, 3D printed PLGA, carbon nanotubes, and mesoporous silica nanoparticles have been used to encapsulate therapeutic agents to be used in implantable controlled drug delivery systems [29, 37, 38]. Of such methods, the distinguishing property of the electropsun scaffolds, is their ability to be tuned and provide various release kinetics of the drug, depending on the selection of the polymer used to fabricate the scaffold [39]. For example, to achieve immediate release of the drug, usually more hydrophilic polymers such as Soluplus, Polyvinyl pyrrolidone (PVP), and combination of Polyvinyl alcohol (PVA) are used to fabricate the scaffold [40–43]. Electrospun fibers can also be manipulated to provide a more sustained release of the drug by using Polycaprolactone (PCL), poly(acrylic acid), PLGA, shellac, silk fibroin or a combination of them [43–47]. Importantly, drug release can also be designed to be externally manipulated using electrospun scaffold when the polymer used has intrinsic properties to be thermo-responsive, pH responsive and electro-responsive; and can also be designed to provide a biphasic release pattern, having an initial immediate release, following with a more sustained one [48–52]. Therefore, encapsulating Honokiol inside of PLGA 85–15 fibers, is the first step to demonstrate a locally deliverable anti-cancer drug for RCC, which not only provides a controlled delivery of a biologically active cancer drug, but also has the potential to be manipulated to release the drug in an on-demand fashion when need be.

As reported in this study, the gradual release of Honokiol from the scaffolds over the time period provides a desired concentration of Honokiol for the cells, which elicits same biological effect as when the cells are incubated with that final cumulative concentration of Honokiol for the entire time period. This proves the advantage of using drug-loaded scaffolds for obtaining the same apoptosis effect while reducing the exposure time of high concentration of toxic cancer drugs to the cells. In the other words, when the release is gradual from the scaffolds, the same apoptosis behavior can be seen for the cells as when cells were incubated with the high concentration of Honokiol for 24 hr (Fig 3). This benefit of Honokiol-loaded scaffolds can reduce the possible undesired side effects for healthy cells that are accompanied by utilization of high concentrations of Honokiol for prolonged time.

In our previous reports, we have demonstrated that Honokiol can inhibit renal tumor growth *in vivo*; and it can also inhibit RTK c-MET-induced growth of renal cancer cells [5]. Importantly, we have reported that Honokiol can also inhibit the expression of tumor cell PD-L1, the ligand for immune checkpoint molecule PD-1; and combination treatments with RTK inhibitors and immune checkpoint inhibitors (like anti-PD-1) are being tested in clinic for RCC [6, 53]. Thus, with potency to decrease PD-L1 expression, Honokiol can be explored as combination therapy with immune checkpoint inhibitors. In this context of using Honokiol with other targeted therapies, gradual and sustained delivery of Honokiol with the help of Honokiol-loaded PLGA 85–15 scaffold implants can be beneficial with favorable therapeutic outcomes.

As discussed earlier, Honokiol can potentially be used as an adjunct immunosuppressive agent for organ transplant recipients and it can prevent immunosuppressive agent-induced growth of renal cancer [5]; and Honokiol-loaded PLGA 85–15 scaffold implant-mediated sustained delivery of Honokiol can be beneficial for these patients. Additionally, depending on the tumor organ or location, Honokiol-loaded scaffolds can also be implanted directly near the tumor site, as wang et.al., reported with a chemotherapeutic agent-loaded PLGA scaffold stent implanted near the tumor in an animal model [54]. For most cancer types, transdermal implants of drug-loaded PLGA scaffolds are ideal and are feasible with PLGA-based microneedle or nanoparticle approaches [55]. Also, PLGA 85–15 is an FDA approved copolymer which is currently being used in clinical studies and provides an appropriate release pattern of Honokiol for the application we desired [56]. Therefore, cytocompatibility and a desired release behavior with a small burst followed by a more sustained release were the reasons behind choosing PLGA copolymer. The degradation byproducts of PLGA 85–15 scaffolds, namely GA and LA are highly acidic in nature and can potentially limit the efficacy of the delivered drugs [57]. However, we have demonstrated that the released Honokiol is biologically active in restricting the growth and migration of renal cancer cells post 24 hr (Fig 3 and Fig 4). However, further investigation of efficacy of the scaffold to be used as long-term source of the drug is needed. A transdermal implant of Honokiol-loaded PLGA 85–15 scaffold can potentially be superior in delivering gradual and sustained doses of Honokiol over 24 hr period of time in replacement for high dose single treatment and the need for treatment with multiple doses. Our results, that Honokiol released from the PLGA 85–15 scaffolds is biologically active, serves as the first step in developing the PLGA-based Honokiol delivery approaches in the treatment of RCC and other cancer types.

## Conclusion

In summary, we performed *in vitro* studies using renal cancer cell lines and demonstrated that controlled delivery and release of Honokiol can be achieved with electrospun Honokiol-loaded PLGA 85–15 scaffolds over 24 hr. We have highlighted the potential benefits of Honokiol-loaded PLGA 85–15 scaffold implants in the treatment of RCC. However, further studies are needed to develop and demonstrate the long-term *in vitro* efficacy of Honokiol-loaded PLGA 85–15 scaffold implants and their applicability *in vivo*.

## Supporting information

S1 File (PDF)
