# Supplementary material for: Novel Honokiol-eluting PLGA-based scaffold effectively restricts the growth of renal cancer cells
Source: PLoS One. 2020 Dec 17;15(12):e0243837. doi: 10.1371/journal.pone.0243837 (PMC7746163; doi:10.1371/journal.pone.0243837)
Supplement: S1 File — (PDF) [file pone.0243837.s001.pdf]

	Area	Mean	Min	Max	Angle	Length
1	5486.968	126.584	75	185.014	-46.169	430.823
2	5791.8	143.97	108.501	175.019	37.185	457.661
3	5639.384	145.683	98.667	217.01	36.254	447.785
4	6249.047	123.381	75.111	160.262	-53.13	493.827
5	5791.8	132.293	73.667	170.917	-30.7	457.364
6	5486.968	111.52	87.429	150.183	42.709	430.823
7	5486.968	129.53	89.2	150.678	41.496	432.079
8	5486.968	129.53	89.2	150.678	41.496	432.079
9	4572.474	141.235	107.876	186.02	29.249	361.391
10	4877.305	133.932	80.889	172.601	-43.668	384.284
11	4877.305	138.402	99.667	175.449	65.095	382.871
12	5029.721	134.632	81.111	189.667	-48.814	396.431
13	5029.721	140.289	83.292	206.5	-65.854	397.796
14	5029.721	121.788	78.333	165.667	-70.463	400.849
15	5486.968	138.309	101.299	165.684	42.709	430.823
16	5486.968	115.792	89.333	167.162	15.255	427.35
17	6249.047	145.433	106.889	195.018	53.13	493.827
18	4724.889	153.477	112.056	186.889	45	372.468
19	5182.137	120.501	73.556	152.954	95.194	412.837
20	4724.889	141.163	110.987	185.72	-57.381	369.913
21	5486.968	140.456	112.216	181.766	16.39	432.079
22	5182.137	120.809	88.556	154.403	54.866	401.862
23	5944.216	149.895	124.297	188.318	-16.991	463.543
24	6249.047	127.024	86.333	169.333	0	493.827
25	5486.968	119.403	70.333	209.722	-6.71	430.823
26	5029.721	139.744	78	196.667	-64.983	397.796
27	5334.553	133.788	79.778	177.533	-54.462	424.806
28	5639.384	136.931	98.889	192.111	90	444.444
29	3810.395	127.154	82.222	165.167	43.264	291.223
30	6096.632	134.453	89.715	169.627	16.144	479.349
31	6249.047	133.315	88.667	188.19	-45	488.864
32	5791.8	120.98	96.111	145.485	38.418	457.661
33	5182.137	120.203	89.963	161.697	0	411.523
34	5791.8	99.221	75.485	140.229	52.524	457.661
35	5486.968	134.857	106.64	179.122	53.13	434.58
36	5486.968	139.169	100.4	194.187	-33.232	429.248
37	6249.047	135.679	67.333	206.333	-42.955	489.418
38	6249.047	147.94	67.333	255	-42.955	489.418
39	6401.463	112.763	73.84	162.545	-9.926	500.639
40	5182.137	104.299	77.744	155.758	75.964	407.22

Figure-1D Control Fibers

PLGA fibers			**Figure-1D Control Fibers**	
430.823	337.029	362.136	Average	
457.661	430.485	426.124	408.195975	
447.785	387.577	419.75		
493.827	366.768	371.383	SD	error
457.364	410.524	386.079	40.6993748	3.71193808
430.823	405.447	425.716		
432.079	388.42	420.578		
432.079	383.903	442.346		
361.391	397.299	383.366		
384.284	414.488	416.422		
382.871	347.539	350.419		
396.431	406.253	364.768		
397.796	417.632	391.67		
400.849	376.448	356.326		
430.823	378.182	375.111	250-300	1
427.35	419.715	359.968	300-350	4
493.827	416.848	377.192	350-400	51
372.468	369.138	392.114	400-450	45
412.837	357.13	396.744	450-500	18
369.913	446.886	318.98	500-550	1
432.079	386.451	364.768		
401.862	376.448	362.855		
463.543	403.291	348.428		
493.827	444.194	397.619		
430.823	445.175	355.103		
397.796	458.683	356.326		
424.806	431.244	374.879		
444.444	410.258	421.404		
291.223	392.329	417.048		
479.349	389.821	393.442		
488.864	369.138	377.883		
457.661	387.577	398.712		
411.523	401.665	351.163		
457.661	383.903	421.404		
434.58	454.864	396.744		
429.248	426.158	354.858		
489.418	429.217			
489.418	475.486			
500.639	373.252			
407.22	436.607			
485.693	450.141			
390.938	454.18			

Count of PLGA fibers	
Row Labels	Total
250-300	1
300-350	4
350-400	51
400-450	45
450-500	18
500-550	1
Grand Total	120

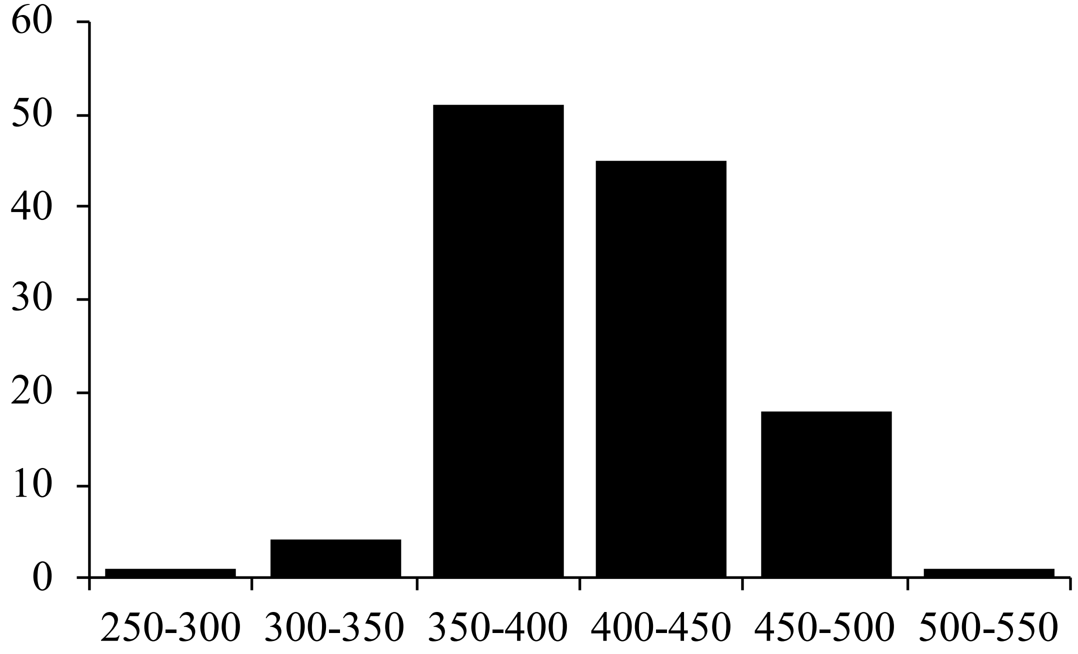

Figure-1D Control Fibers

	Area	Mean	Min	Max	Angle	Length
1	4235.847	142.024	63	244.02	47.386	373.965
2	4477.896	133.73	83.296	193.358	-26.565	393.587
3	3993.799	144.427	96	235.688	60.255	354.775
4	4114.823	120.967	84.185	166.259	0	366.703
5	4840.968	128.143	88.553	224.124	49.236	426.386
6	4719.944	136.459	83	187.383	-80.789	414.877
7	3509.702	133.704	88.483	198.98	45	311.158
8	4114.823	138.278	96.31	190.429	53.471	366.703
9	3993.799	123.029	96	152.667	82.875	354.775
10	4719.944	124.59	72.232	198.269	9.211	417.205
11	3993.799	146.386	84.75	220	-47.49	353.255
12	3993.799	140.497	99.25	220	63.435	347.731
13	4840.968	142.033	71.667	208.054	72.031	431.403
14	3630.726	138.591	89.333	213.4	37.694	313.912
15	4114.823	129.654	82.333	183	40.03	364.349
16	4719.944	132.54	85	207.526	48.24	415.396
17	4719.944	131.795	79.111	191.379	-23.199	414.099
18	4477.896	137.52	68.333	191.704	-93.18	396.311
19	4477.896	117.823	76.753	180.852	-1.591	396.311
20	4235.847	133.79	82	174.667	-69.444	375.974
21	4719.944	121.293	86	182.994	-7.696	414.877
22	4477.896	120.275	66.83	192.248	30.964	394.952
23	4235.847	133.424	88.321	191.79	-6.911	369.334
24	4477.896	149.916	114.593	199.423	75.579	394.952
25	4356.871	112.657	74	166.429	-36.87	387.25
26	4356.871	152.274	81	201.849	81.87	385.858
27	3630.726	149.561	125.605	170.225	-50.44	323.032
28	3993.799	145.203	86	209.802	68.199	348.658
29	3872.775	114.063	85	150.303	-30.651	342.117
30	4477.896	104.363	80.333	139.649	23.806	400.631
31	4477.896	124.234	58.37	168.556	-97.907	400.362
32	4114.823	102.249	69.444	127.376	12.339	359.594
33	4719.944	97.427	73.437	128.492	-13.325	423.348
34	3630.726	133.574	68.333	183.579	-90	322.699
35	4356.871	106.093	73.667	139.741	6.52	383.902
36	4114.823	125.292	79.412	145.646	-77.661	359.594
37	3993.799	120.505	65.667	184.333	42.51	353.255
38	3872.775	119.381	78.778	198.083	-9.162	342.431
39	4356.871	112.389	80.874	159.08	-8.13	385.858
40	4356.871	119.344	69.703	213.677	6.52	383.902

Figure-1D-Honokiol Fibers

PLGA-Honokiol 0.2 **Figure-1D**

373.965	405.033	403.031
393.587	388.873	423.205
354.775	408.723	378.995
366.703	409.057	436.814
426.386	374.572	399.495
414.877	430.832	393.232
311.158	397.894	313.433
366.703	385.702	407.407
354.775	408.723	384.107
417.205	397.894	418.147
353.255	424.77	414.313
347.731	421.221	358.311
431.403	418.295	407.407
313.912	385.702	395.48
364.349	401.989	470.81
415.396	392.715	378.526
414.099	541.194	411.306
396.311	399.605	402.591
396.311	380.357	399.051
375.974	413.041	376.648
414.877	399.605	411.306
394.952	387.114	351.818
369.334	450.953	407.407
394.952	479.128	407.407
387.25	421.221	372.862
385.858	491.224	413.027
323.032	414.361	413.456
348.658	386.762	407.407
342.117	385.348	399.051
400.631	399.264	418.147
400.362	475.697	421.105
359.594	423.16	407.407
423.348	454.57	399.051
322.699	413.371	418.147
383.902	462.901	418.147
359.594	394.103	465.126
353.255	421.221	
342.431	439.923	
385.858	438.03	
383.902	418.147	
408.723	416.873	
413.041	413.027	

Average	
399.6885	
SD	error
35.5080313	3.24142494

Count of PLGA-Honokiol 0.2

Row Labels	Total
300-350	9
350-400	50
400-450	52
450-500	8
500-550	1
Grand Total	120

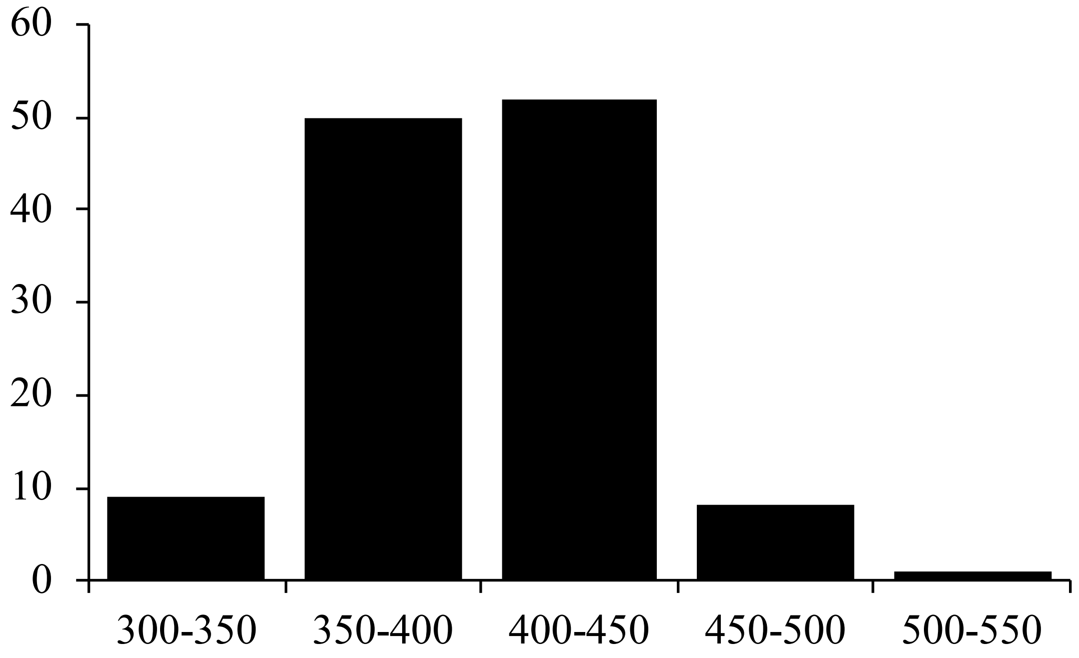

Figure-1D-Honokiol Fibers

Time (minutes)	Concentration	Percentage of HNK released
5	4.07352941	0.354133109
10	6.83823529	0.594483378
15	9.5	0.825884435
30	25.5882353	2.224518448
45	36.4411765	3.1680211
60	42.1911765	3.667898521
90	58.2941176	5.067810993
120	67.7794118	5.892416969
180	93.1470588	8.097758534
240	111.852941	9.723958227
360	149.411765	12.9891422
1440	309.220588	26.88215483
2880	442.240196	38.44624154

Figure-2B
DATA SET 1
Cumulative release of Honokiol in 200 uL

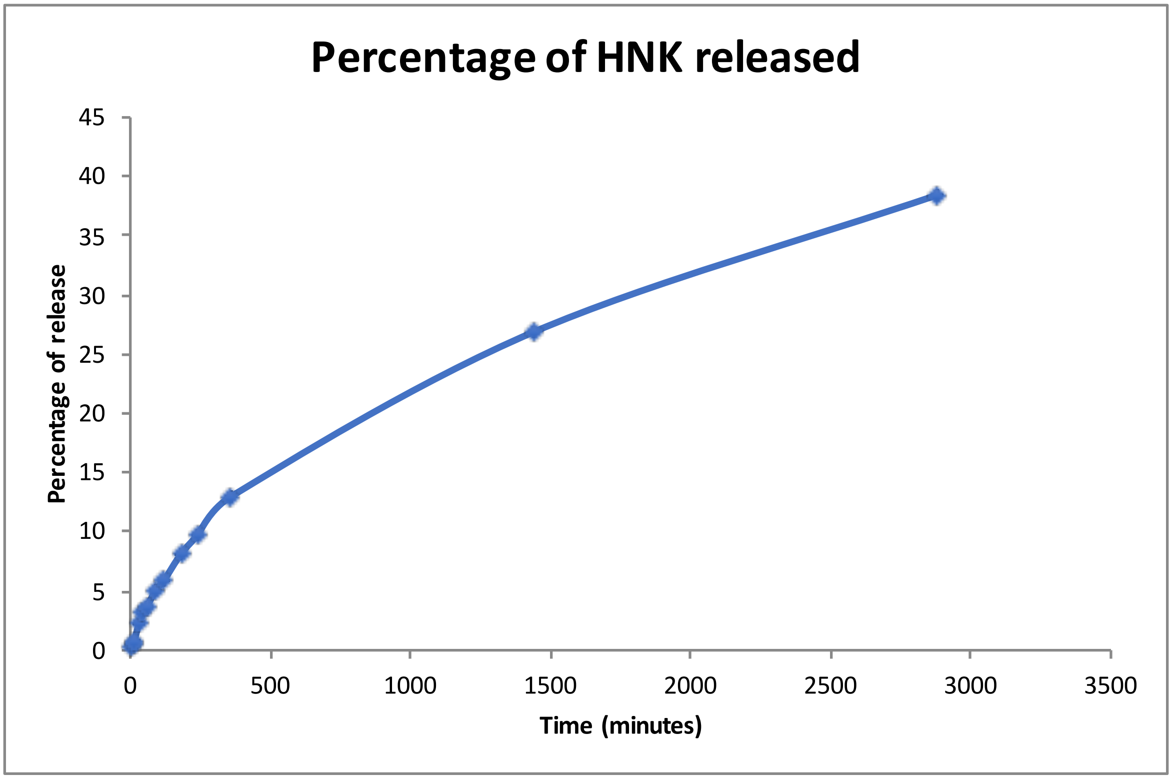

Concentration		Absorbance	SD	Error
0.1	0.171	0.009	0.005508	0.00318
0.5	0.173667	0.0116667	0	0
1	0.1736	0.0116	0.001528	0.000882
2.5	0.1706	0.0086	0.003786	0.002186
5	0.182333	0.0203333	0.005859	0.003383
7.5	0.180667	0.0186667	0.002082	0.001202
10	0.189	0.027	0.004583	0.002646
12.5	0.19	0.028	0.001	0.000577
15	0.199667	0.0376667	0.006429	0.003712
17.5	0.201	0.039	0.002646	0.001528
20	0.204667	0.0426667	0.004041	0.002333

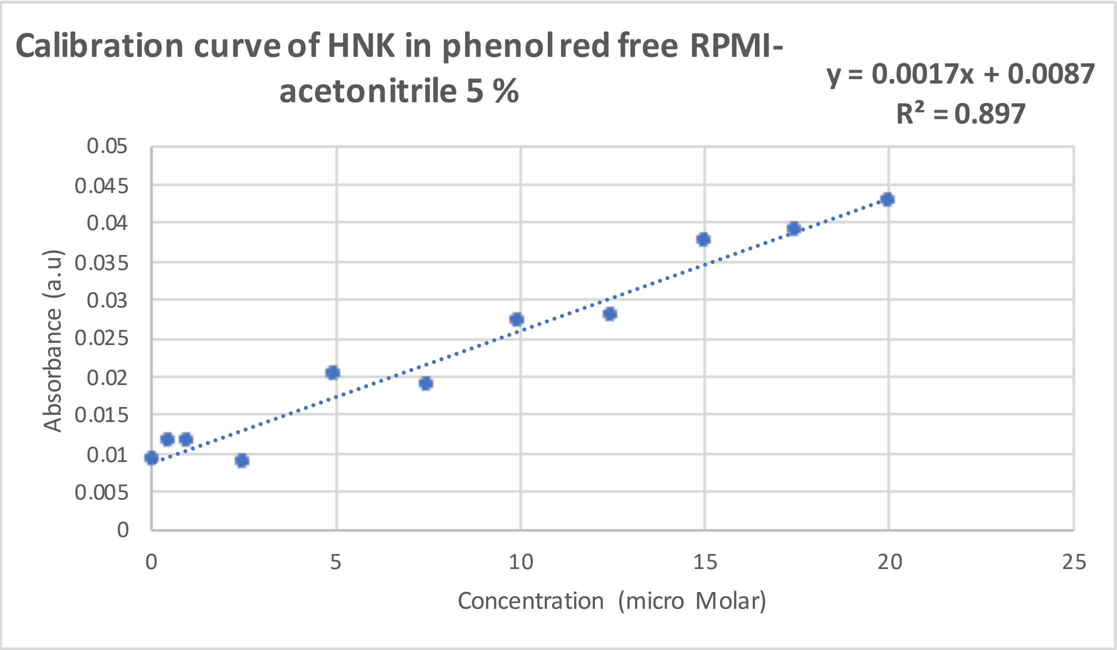

Concentraion/Absorbance Curve for Honokiol (used for Figure 2B)

DATA SET 2

Cumulative release of Honokiol in 200 uL

Time (minutes)	Concentration	Percentage of HNK released
5	1.156862745	0.135021329
10	7.235294118	0.844455429
15	9.588235294	1.119075081
30	17.23529412	2.011588949
45	34.29411765	4.002581425
60	46.45098039	5.421449626
90	55.2745098	6.45127332
120	78.80392157	9.197469838
180	86.05882353	10.04421376
240	113.5098039	13.2481097
360	163.5098039	19.0837773
1440	202.7254902	23.6607715
2880	250.1764706	29.19893448

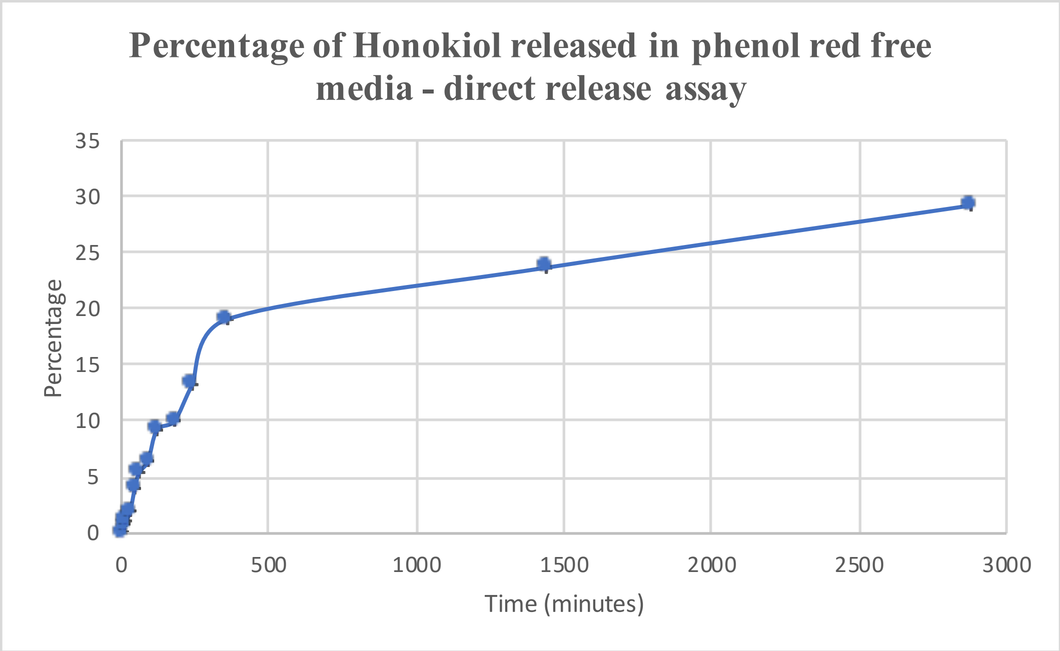

(Additional data to support Figure-2B)

Figure-3A

Apoptotic cells	Late	Early	Total	Average	S.D.
Control	1.29	7.04	8.33		
	0.98	5.02	6	7.165	1.6475588
	1.76	11.94	13.7		
rHNK	1.73	11.74	13.47	13.585	0.16263456
	1.87	9.83	11.7		
Hnk	1.49	10.59	12.08	11.89	0.26870058

Control	7.165	1.6475588
rHNK	13.585	0.16263456
HNK	11.89	0.26870058

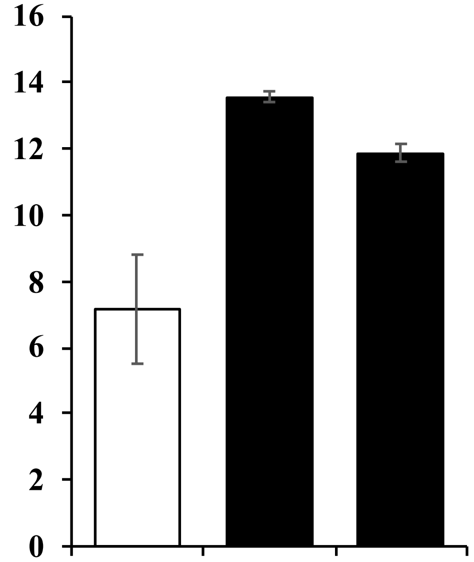

Figure-3B

Apoptotic cells	Late	Early	Total	Average	S.D.
Control	3.36	4.41	7.77		
	3.62	5.62	9.24	8.505	1.03944697
	7.38	10.27	17.65		
rHNK	8.12	8.6	16.72	17.185	0.65760931
	8.82	7.8	16.62		
hnk	5.56	8.66	14.22	15.42	1.69705627

Control	8.505	1.03944697
rHNK	17.185	0.65760931
HNK	15.42	1.69705627

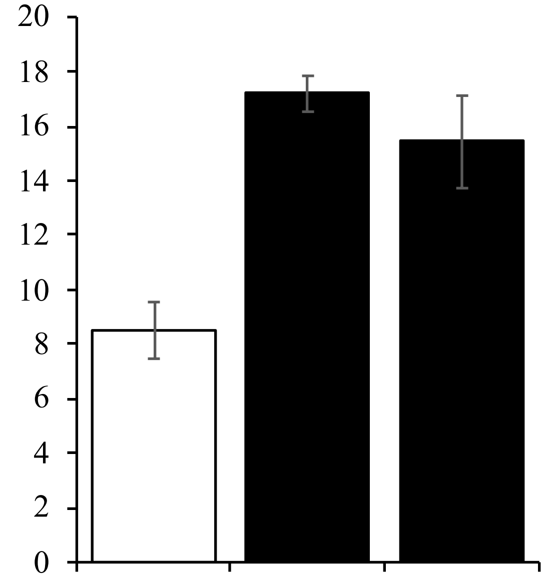

Figure-3C (786-0 cells)

Apoptotic cells	Late	Early	Total	Average	SD
control	2.62	2.42	5.04		
	2.24	3.88	6.12	5.58	0.76367532
	10.96	7.02	17.98		
rHNK	7.16	8.89	16.05	17.015	1.36471609

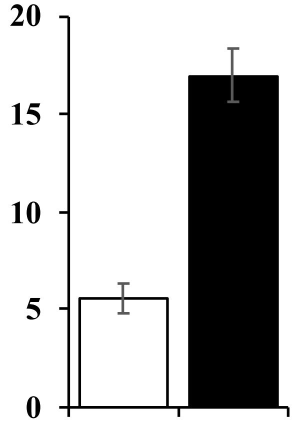

Control	5.58	0.76367532
rHNK	17.015	1.36471609

Figure-3C-ACHN cells

Apoptotic Cells	Early	Late	Total	Average	SD
Control	2.15	1.35	3.5		
	3.02	1.26	4.28	3.89	0.55154329
	14.7	7.1	21.8		
rHNK	13.92	5.32	19.24	20.52	1.81019336

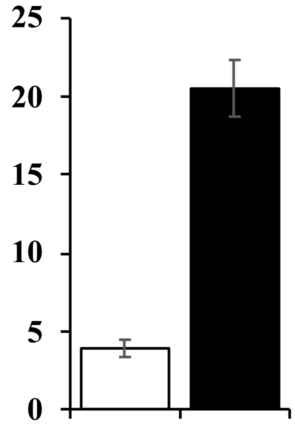

Control	3.89	0.55154329
rHNK	20.52	1.81019336

Figure-4A-786-0 cells

Absorbance

Control	HNK scaffold	
1.3	0.7	
1.19	0.52	
1.31	0.6	
1.26	0.68	
1.2	0.7	
0.05540758	0.082259751	SD

Control	HNK Scaffold	
1.252	0.64	Average

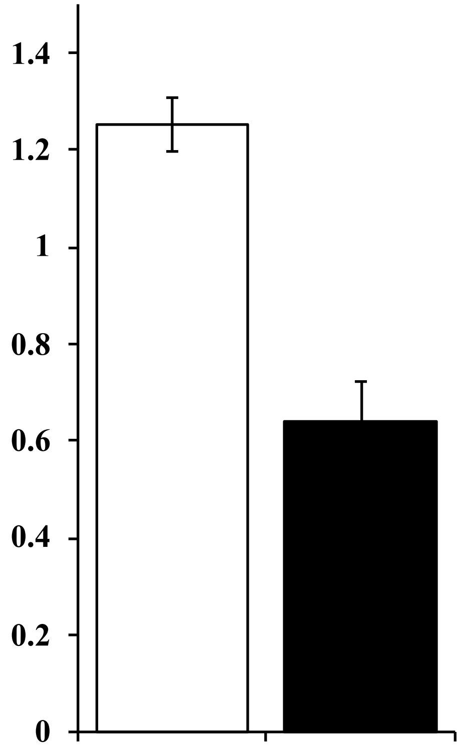

Figure-4A-ACHN cells

Absorbance

control	HNK scaffold	
1	0.49	
1.1	0.54	
1.05	0.58	
1.12	0.53	
0.99	0.55	
0.0580517	0.03696846	SD

control	HNK scaffold	
1.052	0.538	Average

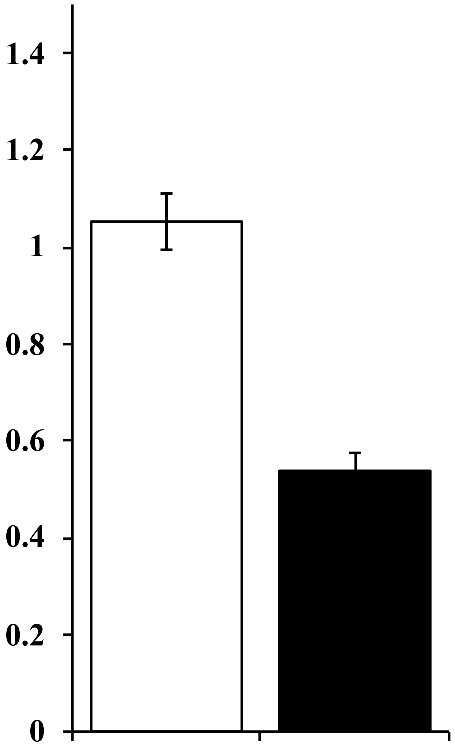

Figure-4C

ACHN	No. of cells migrated	SD
Control	230	7.5
Honokiol fibers	140	4

786-O	No. of cells migrated	SD
Control	285	8.5
Honokiol fibers	136	5

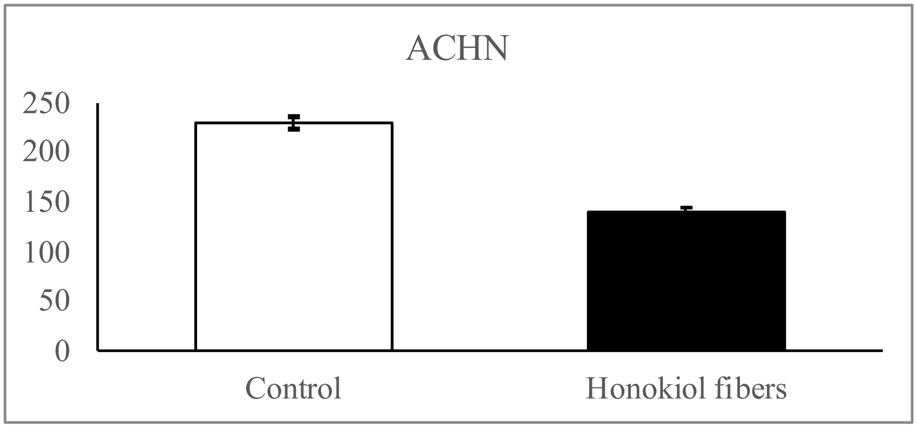

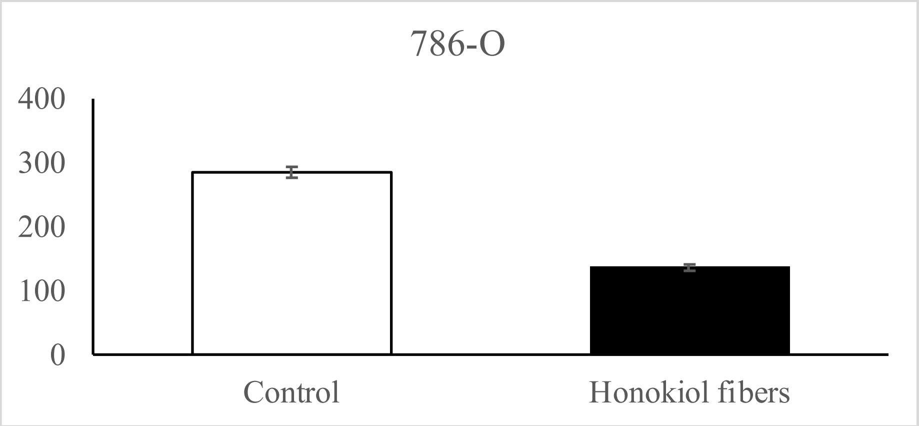

Figure-4C

ACHN	Control	Honokiol fibers
No. of cells migrated	223	144
	229	140
	238	136
Avg	230	140
SD	7.54983444	4

786-O	Control	Honokiol fibers
No. of cells migrated	293	141
	285	136
	276	131
Avg	284.666667	136
SD	8.50490055	5
